# Impact of climate change on “evaporation paradox” in province of Jiangsu in southeastern China

**DOI:** 10.1371/journal.pone.0247278

**Published:** 2021-02-19

**Authors:** Mengsheng Qin, Yuan Zhang, Shiquan Wan, Yuan Yue, Yuan Cheng, Bo Zhang

**Affiliations:** 1 Yangzhou Meteorological Office, Yangzhou, China; 2 Meteorological Observatory of Jilin Province, Changchun, China; Soil and Water Resources Institute ELGO-DIMITRA, GREECE

## Abstract

Contrary to the common expectation that the reference evapotranspiration (ET_o_), which is an indicator of the atmospheric evaporation capability, increases in warming climate, the decline of the ET_o_ has been reported worldwide, and this contradiction between the expected increasing ET_o_ and the observed decreasing one is now termed the “evaporation paradox”. Based on the updated meteorological data (1960–2019), we separately detected the spatiotemporal characteristics and the causes of the “evaporation paradox” in three subregions, namely Huaibei, Jianghuai, and Sunan, and throughout the entire province of Jiangsu in southeastern China. Different from the reported continuous unidirectional variations in the ET_o_, in the province of Jiangsu, it generally showed a decreasing trend before 1990 but followed an increasing trend from 1990 to 2019, which led to the different characteristics of the “evaporation paradox” in the periods from 1960 to 1989, from 1990 to 2019, and from 1960 to 2019. In the first 30 years, the reduction of the wind speed (WS) was the main reason for the decreased ET_o_, which consequently gave rise to the “evaporation paradox” in spring and winter in the Huaibei region and only in winter in the two other subregions and throughout the entire province. We noticed that the “evaporation paradox” in spring in the Sunan region was expressed by the decreased daily mean air temperature (*T*_mean_) and the increased ET_o_ which was chiefly induced by the decreased relative humidity (RH) and the increased vapor pressure deficit (VPD). After 1990, the decreased WS also dominated the decreased ET_o_ and resulted in the “evaporation paradox” in winter in the Jianghuai region. Furthermore, the decreased sunshine hour (SH) was the main factor influencing the decreased ET_o_, thereby inducing the “evaporation paradox” in summer and autumn in the Jianghuai region and only in autumn in the Huaibei region and throughout the whole province from 1990 to 2019. In the whole study period from 1960 to 2019, the decreased SH was also found to be responsible for the decreased ET_o_ and for the “evaporation paradox” in summer in all the subregions and throughout the whole province. However, regarding the “evaporation paradox” in autumn, in winter, and in the entire year in the Huaibei region and throughout the whole province, the observed decreased ET_o_ was largely due to the reduced WS from 1960 to 2019. In summary, in addition to the air temperature, the ET_o_ has shifted due to the other meteorological variables (especially the WS, the SH, and the VPD) and shaped the unique spatiotemporal characteristics of the “evaporation paradox” in the province of Jiangsu in southeastern China. Moreover, future studies and simulations addressing the regional climate change and hydrological cycles should take account of the changeable key meteorological variables in different subregions and seasons of the province of Jiangsu.

## Introduction

It is widely accepted that the global air temperature has risen by 0.85°C in the past five decades and will continue to rise by 0.3–4.8°C by the end of this century [1]. Along with the warming trend, there has been a long-term expectation that the pan evaporation (ET_pan_) or the reference evapotranspiration (ET_o_), which indicates the atmospheric evaporative demand, will increase [2]. However, these variables have been found to decrease in many regions worldwide [3–8] since the first report in 1995 [9]. Furthermore, Roderick et al. [10] defined the inconsistency between the expected increasing ET_pan_ or ET_o_ and the observed decreasing ones as the “evaporation paradox”.

Similar to the findings of decreasing ET_o_ or ET_pan_ which was accompanied by the rising air temperature in other countries such as the USA, Canada, Italy, Australia, Iran, and India [4–6, 11–14], we observed the “evaporation paradox” on different spatial and temporal scales within China such as the Yellow River basin during 1960–2010 [15], the Haihe River basin during 1961–2006 [16], the province of Jilin [17], the province of Jiangxi during 1970–2014 [18], and even the entire China during 1961–2013 [19]. However, the consistency in the changes of air temperature and either the ET_pan_ or the ET_o_, that is, the “no evaporation paradox”, was reported in the province of Yunnan during 1991–2011 [20], in the Heihe River basin during 1994–2010 [21], and in the arid region of northwest China during 1993–2010 [22]. Moreover, Cong et al. [23] and Zhang et al. [24] both found the increased ET_o_ along with the rising air temperature throughout the whole China after 1980. In recent years, based on the updated meteorological data, some studies have presented a view that the ET_o_ has had different change points regionally [25, 26], which may impact the spatiotemporal characteristics of the “evaporation paradox” in different regions of China.

Clarifying the dominant meteorological factors in changing the ET_o_ or the ET_pan_ can also explain the causes of the “evaporation paradox” [23]. Generally, most previous studies have reported that the decreased wind speed (WS) and solar radiation overwhelm the positive effect of the increased air temperature and lead to the decreased ET_o_ or ET_pan_, which consequently causes the “evaporation paradox” [6, 14–19, 27]. However, in the last three decades, the increased ET_pan_ or ET_o_ has been observed on a regional scale and considered to be affected not only by the warming trend [28–30] but also by the increased atmosphere demand to a greater extent [22, 23, 31–34]. Therefore, in addition to the air temperature, the impacts of other changing meteorological variables on the trends of the ET_pan_ or the ET_o_ have been considered to be important factors influencing the formation of the “evaporation paradox” [18, 19, 35]. Moreover, in addition to the works investigating the “evaporation paradox” on annual and large-regional scales [16, 18, 19], some studies in recent years have found the different characteristics of the trends of the ET_o_ and the daily mean air temperature(*T*_mean_) in different seasons and subregions [17, 26].

From summarizing the similar works mentioned above, we can first conclude that the relevant studies in China have mostly focused on the arid region of northern China and on the whole country [15–17, 22–24, 30], demonstrating that there appears to be no “evaporation paradox” in the humid southeastern China, which might be contrary to the fact [25, 26]; second, we can infer that most studies have focused on the general trends without considering the abrupt change point in the long-term ET_o_; third, we can deduce that the radiometric terms—including the sunshine hour (SH), the solar radiation, and the air temperature—and the aerodynamic terms such as the WS and the air humidity have impacted on the ET_o_ to different degrees in each climatic regime and thus have dominated the spatiotemporal characteristics of the “evaporation paradox” [11–19, 29–33]. Therefore, it is necessary to examine the long-term changing trend of the ET_o_ and the main factors contributing to it so as to explore the reasons for the “evaporation paradox” in the humid regions of southeastern China.

In this work, we selected the province of Jiangsu, an important economic and agricultural zone in southeastern China, as our study area and proposed a hypothesis to guide this research: changes in the aerodynamic terms, especially the wind speed, have overwhelmed the positive impacts of the increased air temperature, leading to the decreased ET_o_ and the “evaporation paradox” throughout the province of Jiangsu during 1960–2019. According to this hypothesis, we first calculate the daily ET_o_ on both seasonal and annual scales and merge it at these two scales based on the updated meteorological data from March 1960 to February 2020; second, we separately examine and compare the temporal trends of the *T*_mean_ and the ET_o_ in each subregion and throughout the whole province on both seasonal and annual scales to obtain the spatiotemporal characteristics of the “evaporation paradox”; third, the dominating meteorological variables changing the ET_o_ are determined; finally, the reasons for the formation of the “evaporation paradox” on various spatiotemporal scales are discussed.

## Materials and methods

### Study area and data source

**Fig 1:**
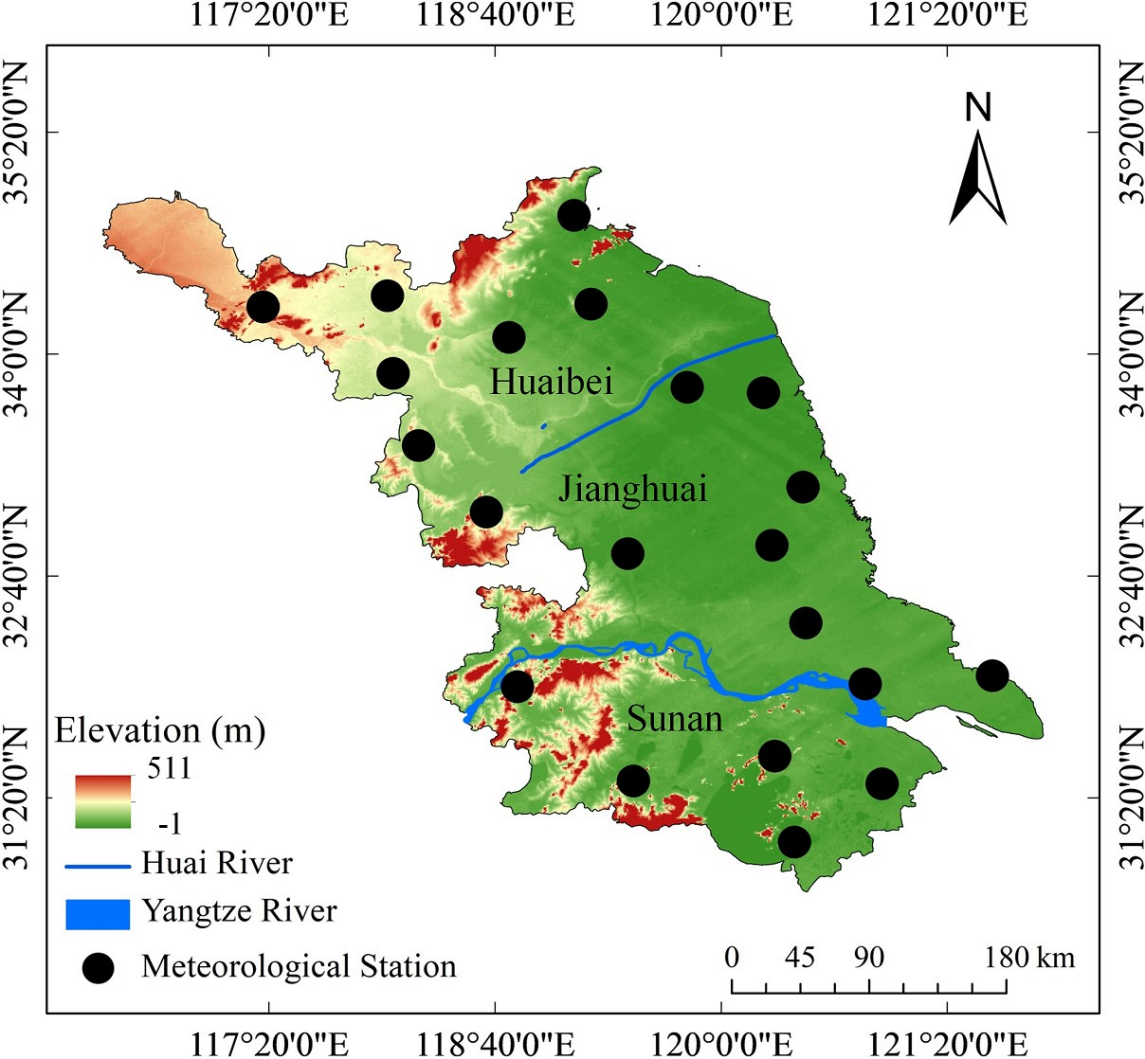
The distributions of the subregions (Huaibei, Jianghuai, and Sunan), the rivers, and the meteorological stations.

The province of Jiangsu is located on the southeastern coast of China (30°45′–35°20′ N, 116°18′–121°57′ E) and covers an area of 1.03×10^5^ km^2^ extending 460 km from north to south and 320 km from east to west. As shown in Fig 1, the Yangtze River and the Huai River divide this study area into the Huaibei region (the north of the Huai River), the Jianghuai region (between the Yangtze River and the Huai River), and the Sunan region (the south of the Yangtze River); the Huaibei region belongs to temperate semi-humid monsoon climate, and the latter two subregions both belong to subtropical humid monsoon climate [36, 37]. The multiyear average *T*_mean_ in the Huaibei, Jianghuai, and Sunan subregions ranges from 13 to 14°C, from 14 to 15°C, and from 15 to 16.5°C, respectively [38]. Furthermore, the differences in the climate and the position of the province of Jiangsu have caused the annual precipitation to range from 800 mm in the northwest to 1200 mm in the southeast, and nearly 60% of the precipitation is concentrated in summer, which is mainly induced by the summer monsoon from the low-latitude ocean [38]. In this study, we discuss the characteristics and the causes of the “evaporation paradox” in the three subregions and throughout the whole province separately.

The newest meteorological dataset by the end of February 2020 was collected from the China Meteorological Data Service Center (http://data.cma.cn/). We extracted the daily data, including the wind speed (m·s^−1^), the relative humidity (%), the sunshine hour (h), the mean air temperature (*T*_mean_, °C), the maximum air temperature (*T*_max_, °C), and the minimum air temperature (*T*_min_, °C) of 21 meteorological sites within the province of Jiangsu during 1960–2019 (Fig 1). Although these variables were preprocessed by China Meteorology Administration, we found the missing daily data (<0.1% in each site) and replaced them with the data on a nearby site. Moreover, we analyzed the data in spring (March–May), in summer (June–August), in autumn (September–November), in winter (December–February in the next year), and in the entire year.

### FAO 56 Penman-Monteith method for estimating ET_o_

The FAO 56 Penman–Monteith (FAO 56 P–M) model, incorporating both the radiometric terms and the aerodynamics terms, has been recommended as the sole standard method for estimating the ET_o_ by the Food and Agriculture Organization of the United Nations [39] and has been preferred well in many regions worldwide [4, 5, 26, 32–34]. The FAO 56 P–M model can be expressed by [39]:

$${ET}_{o}=\frac{0.408\Delta \left(R_{n}-G\right)+\gamma \frac{900}{T+273}U_{2}(e_{s}-e_{a})}{\Delta +\gamma (1+0.34U_{2})}$$ (1)

where *Δ* is the slope of the saturated vapor pressure curve (kPa·°C^−1^), *R*_*n*_ stands for the net radiation (MJ·m^−2^·day^−1^), *G* represents the soil heat flux density, which is zero on a daily scale, (MJ·m^−2^·day^−1^), *γ* indicates the psychrometric constant (kPa·°C^−1^), *T* is the mean daily air temperature (°C), *U*_2_ represents the mean daily wind speed at a height of 2 m (m·s^−1^), *e*_*s*_ denotes the saturated vapor pressure (kPa), *e*_*a*_ is the actual vapor pressure (kPa), and *e*_*s*_−*e*_*a*_ indicates the vapor pressure deficit (VPD) (kPa).

The slope of the saturated vapor pressure curve is calculated as a function of the mean daily air temperature:

$$\Delta =\frac{44098[0.6108\;exp(\frac{17.27T_{mean}}{T_{mean}+237.3})}{{\left(T_{mean}+237.3\right)}^{2}}$$ (2)

The net radiation is also calculated by a series of operations based on the observed SH, *T*_max_, and *T*_min_:

$$R_{n}=R_{ns}-R_{nl}$$ (3)

$$R_{ns}=(1-\alpha )R_{s}$$ (4)

$$R_{s}=\left(a+b\frac{n}{N}\right)R_{a}$$ (5)

$$R_{a}=\frac{1440}{\pi }G_{sc}d_{r}\omega_{s}sin(\varphi )sin(\delta )+cos(\varphi )cos(\delta )sin(\omega_{s})$$ (6)

$$R_{nl}=\sigma \left(\frac{T_{max,k}^{4}+T_{min,k}^{4}}{2}\right)(0.34-0.14\sqrt{e_{a}})(1.35\frac{R_{s}}{R_{so}}-0.35)$$ (7)

where *R*_*ns*_ and *R*_*nl*_ are the incoming net shortwave and the outgoing net longwave radiation respectively (MJ·m^−2^·d^−1^); α denotes the albedo and is set at 0.23; *R*_*s*_ indicates the solar radiation (MJ·m^−2^·d^−1^); *n* and *N* are the actual and the maximum possible sunshine hour, respectively (h); *R*_*a*_ stands for the extraterrestrial radiation (MJ·m^−2^·d^−1^); *G*_*sc*_ represents the solar constant (0.08232 MJ·m^−2^·min^−1^); *d*_*r*_ is the inverse relative distance from the earth to the sun; ω_s_ indicates the sunset hour angle (rad); *φ* is the latitude (rad); δ is the solar declination (rad); σ represents the Stefan–Boltzmann constant (4.903×10^−9^ MJ·m^−2^·d^−1^); *T*_max,*k*_ and *T*_min,*k*_ stand for the maximum and minimum absolute temperature within 24 hours respectively (K); *R*_*so*_ is the clear sky solar radiation (MJ·m^−2^·d^−1^); *a* and *b* are the empirical coefficients equal to 0.25 and 0.5, respectively [39].

The psychrometric constant is obtained from the measured atmospheric pressure (*P*, kPa) as follows:

$$\gamma =\frac{c_{p}P}{\varepsilon \lambda }=0.665\times 10^{-3}P$$ (8)

where λ is the latent heat of vaporization (2.45 MJ·kg^–1^), *c*_*p*_ indicates the specific heat at constant pressure (1.013 × 10^−3^ MJ·kg^–1^°C^−1^), and ε represents the ratio of the molecular weight of water vapor to dry air, being equal to 0.622.

The vapor pressure deficit is calculated by the following equations based on the observed *T*_max_, *T*_min_, and RH:

$$VPD=e_{s}-e_{a}$$ (9)

$$e_{s}=\frac{e^{0}(T_{max})+e^{0}(T_{min})}{2}$$ (10)

$$e^{0}(T_{max})=0.6108\;\exp\left[\frac{17.27T_{max}}{T_{max}+273.3}\right]$$ (11)

$$e^{0}(T_{min})=0.6108\;\exp\left[\frac{17.27T_{min}}{T_{min}+273.3}\right]$$ (12)

$$e_{a}=e_{s}\times RH$$ (13)

More details about calculating the variables required to estimate the ET_o_ can be found elsewhere [26, 39].

### Statistical test for trend analysis

In this study, the widely used nonparametric Mann–Kendall (MK) test [40, 41] and Theil–Sen estimator [42, 43] were separately utilized to examine the changing trend and the slope (rate) of the trend of each variable. The significance of each changing trend was also determined by the nonparametric MK test. Moreover, we used the nonparametric Pettitt test [44] to identify the abrupt change points of the meteorological variables and the ET_o_ series on both seasonal and annual scales. More details about these methods and their applications can be found in references [32, 33, 45–49].

### Detrending method

Herein, we adopted a simple but useful detrending method which has worked successfully within China [3, 26, 32, 46, 48, 50] to quantify the effects of the changing meteorological variables on the trends of the ET_o_. This method consists of the following three steps: (i) removing the changing trends of the WS, RH, VPD, SH, *T*_mean_, *T*_max_, and *T*_min_ to render them stationary; (ii) recalculating the ET_o_ using one detrended variable while maintaining the other variables unchanged; and (iii) separately comparing each recalculated ET_o_ with the original ET_o_ and presenting an evaluating indicator (*R*) defined as:

$$R=\sum_{i=1}^{m}\frac{\left({ET}_{o}^{o}-{ET}_{o}^{R}\right)}{{ET}_{o}^{o}i}$$ (14)

where ${ET}_{o}^{o}$ and ${ET}_{o}^{R}$ are the original and recalculated ET_o_ respectively, and *m* is the length of the data set. A positive, negative, or zero value of *R* separately indicates that the variation in this climatic factor has positive, negative, or no effects on the changes of ET_o_. A larger value of |R| denotes that the variation in this climatic factor affects the ET_o_ to a greater extent [26, 48, 50].

## Results

### Analysis of basic meteorological variables

The results of the Pettitt test showed that the abrupt change points of all the annual meteorological variables in the different subregions and throughout the entire province mostly appeared almost in 1990 (Table 1). Therefore, we divided the entire study period into two 30-year periods; the World Meteorological Organization (WMO) also recommends that a 30-year period should be used as a reference to describe the climate state. Further analyses were thus carried out in three periods: from 1960 to 1989, from 1990 to 2019, and from 1960 to 2019 (Table 1).

**Table 1:** Analysis of the annual meteorological variables in the Huaibei (HB), Jianghuai (JH), and Sunan (SN) regions and throughout the entire province of Jiangsu using Mann–Kendall test, Theil–Sen estimator, and Pettitt test.

Zone	Variables	Values			Trends			Change
		1960–1989	1990–2019	1960–2019	1960–1989	1990–2019	1960–2019	point
HB	T_mean_(°C)	13.84	14.80	14.32	–0.001	0.038**	0.028***	1991
	T_max_(°C)	19.15	19.76	19.46	–0.021^+^	0.029*	0.017**	1992
	T_min_(°C)	9.3	10.71	10.01	0.020^+^	0.045**	0.043***	1991
	WS(m/s)	2.18	1.48	1.83	–0.041***	–0.003***	–0.023***	1988
	VPD(kPa)	0.49	0.54	0.51	–0.001	0.004**	0.0002***	1992
	RH(%)	73.39	71.94	72.66	0.031	–0.098*	–0.044*	1993
	SH(h)	6.48	5.79	6.14	–0.015	–0.029**	–0.023***	1995
JH	T_mean_(°C)	14.52	15.40	14.91	–0.001	0.038**	0.029***	1992
	T_max_(°C)	19.21	19.93	19.52	0.020*	0.032*	0.023***	1992
	T_min_(°C)	10.60	11.78	11.19	0.008	0.039**	0.037***	1990
	WS(m/s)	2.47	2.03	2.25	–0.019***	–0.016***	–0.016***	1988
	VPD(kPa)	0.39	0.47	0.43	–0.001	0.003**	0.0002***	1992
	RH(%)	78.81	76.66	77.74	–0.007	–0.102*	–0.064***	1991
	SH(h)	6.00	5.61	5.81	–0.017*	–0.018*	–0.014***	1995
SN	T_mean_(°C)	15.43	16.67	16.05	–0.011*	0.050***	0.027***	1992
	T_max_(°C)	19.86	20.95	20.41	–0.018*	0.041**	0.029***	1992
	T_min_(°C)	11.85	13.25	12.55	0.003	0.056***	0.044***	1988
	WS(m/s)	2.39	1.87	2.13	–0.018***	–0.015***	–0.017***	1988
	VPD(kPa)	0.4	0.56	0.48	–0.001	0.007***	0.004***	1990
	RH(%)	79.89	75.25	77.57	–0.013	–0.229**	–0.135***	1991
	SH(h)	5.68	5.12	5.40	–0.019**	–0.005	–0.009***	1995
Whole	T_mean_(°C)	14.45	15.48	14.96	–0.004	0.041**	0.028***	1991
	T_max_(°C)	19.30	20.12	19.71	–0.021*	0.036**	0.023***	1992
	T_min_(°C)	10.46	11.77	11.12	0.010	0.045**	0.040***	1992
	WS(m/s)	2.35	1.81	2.08	–0.026***	–0.011***	–0.018***	1990
	VPD(kPa)	0.43	0.51	0.47	–0.001	0.004***	0.003***	1992
	RH(%)	77.26	74.75	76.01	0.008	–0.128*	–0.074***	1993
	SH(h)	6.09	5.54	5.82	–0.016*	–0.018*	–0.016***	1995

Note: ***,**,*, and ^+^indicate the significance level of 0.001, 0.01, 0.05, and 0.1 respectively.

#### Characteristics of basic meteorological variables

During 1960–1989, we found that the Sunan region had the highest annual air temperature variables, that is, a *T*_mean_, *T*_max_, and *T*_min_ of 15.43, 19.86, and 11.85°C, while the Huaibei region had the lowest *T*_mean_, *T*_max_, and *T*_min_ of 13.84, 19.15, and 9.3°C, respectively (Table 1). The entire province during 1960–1989 had an annual *T*_mean_, *T*_max_, and *T*_min_ of 14.45, 19.3, and 10.46°C, respectively (Table 1). Different from the air temperature variables, the maximum annual wind speed during 1960–1989 was found in the Jianghuai region (2.47 m·s^−1^) followed by the Sunan region, the entire province, and the Huaibei region with a wind speed of 2.39, 2.35, and 2.18 m·s^−1^, respectively. The air humidity increased in the three subregions from north to south; in fact, the annual RH increased from 73.39% in the Huaibei region to 79.89% in the Sunan region, but the annual VPD rose from 0.4 kPa in the Sunan region to 0.49 kPa in the Huaibei region (Table 1). Furthermore, the annual sunshine hour in the entire province of Jiangsu was 6.09 h with the longest sunshine of 6.48 h in the Huaibei region and the shortest sunshine of 5.68 h in the Sunan region (Table 1).

Compared to the variables during 1960–1989, the annual air temperature variables enlarged in the second 30-year period (1990–2019) with the lower air humidity expressed by the higher VPD and the lower RH in the province of Jiangsu (Table 1). Moreover, the annual wind speed and sunshine hour were both found to be lower during 1990–2019 than during 1960–1989 (Table 1). Specifically, the annual *T*_mean_, *T*_max_, and *T*_min_ separately varied from 14.8, 19.76, and 10.71°C in the Huaibei region to 16.67, 20.95, and 13.25°C in the Sunan region during 1990–2019; also, the annual *T*_mean_, *T*_max_, and *T*_min_ were 15.48, 20.12, and 11.77°C respectively in the entire province (Table 1). Further, according to Table 1, the maximum annual wind speed of 2.03 m·s^−1^ was found in the Jianghuai region, and the annual WS in the Huaibei region, in the Sunan region, and throughout the entire province was 1.48, 1.87, and 1.81 m·s^−1^ respectively, all being lower than 2 m·s^−1^. The VPD and the RH, the variables related to air humidity, were in the range of 0.47 kPa (Jianghuai) to 0.56 kPa (Sunan) and in the range of 71.94% (Huaibei) to 76.66% (Jianghuai) respectively; also, the VPD and the RH were 0.51 kPa and 74.75% respectively in the entire province during 1990–2019 (Table 1). Moreover, in the second 30-year period, the annual sunshine hour of the entire province was 5.54 h with the longest sunshine in the Huaibei region (5.79 h) followed by the Jianghuai region (5.61) and the Sunan region (5.12 h).

Table 1 also demonstrates that, for the entire study period from 1960 to 2019, the Huaibei region had the lowest annual *T*_mean_, *T*_max_, and *T*_min_ of 14.32, 19.46, and 10.01°C respectively, and the highest annual *T*_mean_, *T*_max_, and *T*_min_—respectively equal to 16.05, 20.41, and 12.55°C—were observed in the Sunan region. Furthermore, the annual wind speed during 1960–2019 ranged from 1.83 m·s^−1^ in the Huaibei region to 2.25 m·s^−1^ in the Jianghuai region; the WS of the whole province was 2.08 m·s^−1^. The driest atmosphere was also found in the northern Huaibei region with an annual VPD and RH of 0.51 kPa and 72.66%, respectively. However, the annual VPD in the Jianghuai and Sunan regions was 0.43 and 0.48 kPa respectively, and the Jianghuai and Sunan regions had relative humidity of 77.74% and 77.57% respectively. The annual sunshine hour of the Huaibei, Jianghuai, and Sunan subregions was 6.14, 5.81, and 5.4 h respectively, indicating a declining trend from north to south and leading to an annual SH of 5.82 h for the entire province during 1960–2019.

#### Temporal trends of basic meteorological variables

As Table 1 reveals, except for the annual *T*_max_ of the Jianghuai region, which increased significantly by 0.02°C·yr^–1^ (*p*< 0.05), the annual *T*_mean_ and *T*_max_ both showed a decreasing trend in all the subregions and the entire province during 1960–1989 with the rate of reduction in the *T*_mean_ ranged from –0.011°C·yr^–1^ in the Sunan region (*p*< 0.05) to –0.001°C·yr^–1^ in the Huaibei region, and the rate of reduction in the *T*_max_ varied from –0.021°C·yr^–1^ in the Huaibei region (*p*< 0.1) to –0.018°C·yr^–1^ in the Sunan region (*p*< 0.05). The annual *T*_min_ increased in this province in the first 30-year period at a rate ranging from 0.003°C·yr^–1^ in the Sunan region to 0.02°C·yr^–1^ in the Jianghuai region (*p*< 0.1). In addition, during 1960–1989, the wind speed was the only meteorological variable which showed a significant decreasing trend in all the subregions and throughout the entire province (*p*< 0.001); it ranged from –0.041 m·s^–1^·yr^–1^ in the Huaibei region to –0.018 m·s^–1^·yr^–1^ in the Sunan region. However, the VPD and RH, both indicating air humidity, showed an insignificant changing trend in the province of Jiangsu during 1960–1989. Specifically, the annual VPD decreased by –0.001 kPa·yr^–1^ in all the subregions and throughout the entire province; the annual RH declined by –0.007%·yr^–1^ and –0.013%·yr^–1^ in the Jianghuai and Sunan regions respectively, but it increased by 0.031%·yr^–1^ and 0.008%·yr^–1^ in the Huaibei region and in the entire province respectively. Further, similar to the wind speed, the annual sunshine hour followed a significant decreasing trend (*p*< 0.05) in the Jianghuai region (–0.017 h·yr^–1^), in the Sunan region (–0.019 h·yr^–1^), and in the entire province (–0.016 h·yr^–1^) but showed an insignificant trend in the Huaibei region (–0.015 h·yr^–1^) during 1960–1989.

As Table 1 presents, after 1990, the annual air temperature variables increased significantly (*p*< 0.05) in this province with rates of 0.038 (Huaibei and Jianghuai)–0.05°C·yr^-1^ (Sunan) for T_mean_, 0.029 (Huaibei)–0.041°C·yr^-1^ (Sunan) for T_max_ and 0.039 (Jianghuai)–0.056°C·yr^-1^ (Sunan) for T_min_, respectively (Table 1). Furthermore, similar to the changing trend of the wind speed during 1960–1989, the annual wind speed during 1990–2019 followed a significant decreasing trend (*p*< 0.001) but at a smaller rate from –0.016 m·s^–1^·yr^–1^ in the Jianghuai region to –0.003 m·s^–1^·yr^–1^ in the Huaibei region. Moreover, different from the insignificant changing trend of the VPD and the RH in the first 30-year period, during 1990–2019, the annual VPD increased significantly at a rate from 0.003 kPa·yr^–1^ in the Jianghuai region (*p*< 0.01) to 0.007 kPa·yr^–1^ in the Sunan region (*p*< 0.001); the annual RH declined significantly at a rate from –0.229%·yr^–1^ in the Sunan region (*p*< 0.01) to –0.098%·yr^–1^ in the Huaibei region (*p*< 0.05). In addition, except for the Sunan region, we observed a significant decreasing trend (*p*< 0.05) of the annual sunshine hour at a rate of –0.029 h·yr^–1^ in the Huaibei region and –0.018 h·yr^–1^ in both the Jianghuai region and the entire province during 1990–2019.

According to Table 1, generally, except for the annual wind speed with greater changes during 1960–1989, the other variables changed more remarkably during 1990–2019, and the changes in these variables dominated in the whole study period from 1960 to 2019. Specifically, the annual *T*_mean_, *T*_max_, and *T*_min_ increased significantly (*p*< 0.001) at the rate separately ranged from 0.027 in the Sunan region to 0.029°C·yr^–1^ in the Jianghuai region, from 0.017 in the Huaibei to 0.029°C·yr^–1^ in the Sunan region and from 0.037 in the Jianghuai region to 0.044°C·yr^–1^in the Sunan region during 1960–2019. Moreover, contrary to the increased air temperature variables, the annual wind speed decreased significantly (*p*< 0.001) at a rate from –0.023 m·s^–1^·yr^–1^ in the Huaibei region to –0.016 m·s^–1^·yr^–1^ in the Jianghuai region during 1960–2019. Furthermore, the significant changing trend (*p*< 0.05) of the VPD and the RH during 1960–2019 was consistent with that during 1990–2019; nonetheless, Table 1 demonstrates that the vapor pressure deficit increased at a smaller rate of 0.0002 kPa·yr^–1^ in the Huaibei and Jianghuai regions and of –0.004 kPa·yr^–1^ in the Sunan region; also, the relative humidity declined at a rate from –0.135%·yr^–1^ in the Sunan region to –0.044%·yr^–1^ in the Huaibei region. Finally, in the whole study period, the annual sunshine hour followed a significant negative trend (*p*< 0.001) in the province of Jiangsu with the minimum rate of –0.009 h·yr^–1^ in the Sunan region and the maximum rate of –0.023 h·yr^–1^ in the Huaibei region.

### Analysis of seasonal and annual ET_o_

As listed in Table 2, the change points of the seasonal and annual ET_o_ in each subregion and throughout the entire province all appeared during 1988–1995. Combined with the change points of the basic meteorological variables seen almost in 1990, we separately analyzed the seasonal and annual ET_o_ in the three study periods: from 1960 to 1989, from 1990 to 2019, and from 1960 to 2019.

**Table 2:** Analysis of the seasonal and annual ET_o_ estimated by the FAO-56 Penman–Monteith model in the Huaibei (HB), Jianghuai (JH), and Sunan (SN) regions and throughout the entire province of Jiangsu using Mann–Kendall test, Theil–Sen estimator, and Pettitt test.

Zone	Season	Values			Trends			Change
		1960–1989	1990–2019	1960–2019	1960–1989	1990–2019	1960–2019	point
HB	Spring	297.4	292.4	294.9	–0.61	0.95^+^	0.04	1987
	Summer	402.2	375.1	388.7	–1.48**	–0.11	–0.82***	1984
	Autumn	204.8	197.9	201.3	–0.3	–0.46	–0.28**	1985
	Winter	99.3	94.9	97.2	–0.83**	0.06	–0.16*	1992
	Annual	1003.7	960.4	982.1	–3.94***	0.66	–1.37***	1988
JH	Spring	264.9	282.5	273.7	0.36	1.21*	0.65***	1996
	Summer	377.2	365.1	371.1	–1.54**	–0.01	–0.44*	1992
	Autumn	200.3	202.3	201.3	–0.48^+^	–0.44	0.01	1986
	Winter	105.9	110.4	108.2	–0.01	–0.11	0.09	1991
	Annual	948.3	960.3	954.3	–2.44**	0.59	0.08	1992
SN	Spring	264.1	296.8	280.6	0.2	1.94**	1.04***	1991
	Summer	438.1	438.3	438.2	–1.47*	0.97	–0.015	1994
	Autumn	221.1	233.8	227.5	–0.15	0.17	0.36**	1990
	Winter	102.8	106.2	104.5	–0.23	0.15	0.09	1993
	Annual	1026.4	1075.1	1050.8	–2.12*	3.02*	1.32*	1991
Whole	Spring	275.6	289.2	282.4	0.008	1.35*	0.53**	1992
	Summer	400.0	385.9	392.9	–1.45***	0.17	–0.53*	1990
	Autumn	206.7	208.3	207.5	–0.3	–0.31	–0.05	1993
	Winter	103.2	104.3	103.7	–0.32	0.05	–0.01	1992
	Annual	985.4	987.7	986.5	–3.1**	0.98	–0.15	1991

Note: ***,**,*, and ^+^indicate the significance level of 0.001, 0.01, 0.05, and 0.1 respectively.

#### Characteristics of seasonal and annual ETo

Table 2 demonstrates that a higher ET_o_ was found in the Huaibei region in spring: 297.4 and 294.9 mm during 1960–1989 and 1960–2019 respectively; however, a higher ET_o_ (296.8 mm) was found in the Sunan region in spring during 1990–2019. In summer, we observed a higher ET_o_ in the Sunan region, with all the values being almost equal to 438 mm in the three study periods. Moreover, the southern Sunan region had the largest values of ET_o_ in autumn: 221.1 mm during 1960–1989, 233.8 mm during 1990–2019, and 227.5 mm during 1960–2019 (Table 2). In winter, the northern Huaibei region had the lowest ET_o_ values (all lower than 100 mm) in the three study periods; nevertheless, the Jianghuai region had the highest ET_o_ in winter: 105.9 mm during 1960–1989, 110.4 mm during 1990–2019, and 108.2 mm during 1960–2019. On an annual scale, except for the annual ET_o_ of 1003.7 mm in the Huaibei region during 1960–1989, the ET_o_ was lower than 1000 mm in both the Huaibei and Jianghuai regions in the three study periods. Moreover, the annual ET_o_ of the southern Sunan region was higher than 1000 mm: 1026.4 mm during 1960–1989, 1075.1 mm during 1990–2019, and 1050.8 mm during 1960–2019. Table 2 also shows that the annual ET_o_ of the entire province of Jiangsu was around 986 mm: 985.4 mm during 1960–1989, 987.7 mm during 1990–2019, and 986.5 mm during 1960–2019. Generally, the ET_o_ values of the subregions and the entire province all followed the order of summer > spring >autumn > winter during all the three study periods. However, regarding the regional differences in the ET_o_ in the same season, a higher ET_o_ in spring was found in the Sunan region during 1990–2019 and in the Huaibei region during both 1960–1989 and 1960–2019. A higher ET_o_ was found in the Sunan region in summer, in autumn, and throughout the entire year, but a higher ET_o_ was observed in the Jianghuai region in winter during all the three study periods (Table 2).

#### Temporal trends of seasonal and annual ETo

During 1960–1989, the spring ET_o_ showed an insignificant changing trend with a negative rate of –0.61 mm·yr^–1^ in the northern Huaibei region and a positive rate of 0.36, 0.2, and 0.008 mm·yr^–1^ in the Jianghuai region, the Sunan region, and the entire province, respectively (Table 2). For the other seasons during 1960–1989, the ET_o_ of each subregion and the entire province showed a decreasing trend with the highest rate of reduction seen in summer; the rate of reduction in the ET_o_ ranged from –1.54 mm·yr^–1^ in the Jianghuai region (*p*< 0.01) to –1.47 mm·yr^–1^ in the Sunan region (*p*< 0.05). Furthermore, the lowest rate of reduction in the ET_o_ during 1960–1989 was observed in autumn with a value of –0.15 mm·yr^–1^ in the Sunan region and –0.3 mm·yr^–1^ in both the Huaibei region and the entire province. Also, the Jianghuai region showed the lowest rate of reduction in the ET_o_ (–0.01 mm·yr^–1^) in winter during 1960–1989 (Table 2). In addition, on an annual scale in the first 30-year period, the annual ET_o_ in the Huaibei region declined sharply at a rate of –3.94 mm·yr^–1^ (*p*< 0.001), and the annual ET_o_ decreased at a lower rate of –2.44 mm·yr^–1^ (*p*< 0.01) and –2.12 mm·yr^–1^ (*p*< 0.05) in the Jianghuai and Sunan regions, respectively. Moreover, the annual ET_o_ of the entire province followed a significant decreasing trend (*p*< 0.01) with a rate of –3.1 mm·yr^–1^ during 1960–1989.

According to Table 2, in the second 30-year period, there was a slight decrease in the ET_o_ (insignificant and lower than 0.5 mm·yr^–1^) in summer in the Huaibei and Jianghuai regions; in autumn in the Huaibei region, in the Jianghuai region, and throughout the entire province; and in winter only in the Jianghuai region. However, in the other regions and seasons, the ET_o_ exhibited an increasing trend with the highest rate in spring; the rate of increase in the ET_o_ ranged from 0.95 mm·yr^–1^ in the Huaibei region (*p*< 0.1) to 1.94 mm·yr^–1^ in the Sunan region (*p*< 0.01). The lowest rate of increase in the ET_o_ was observed in winter with only 0.06, 0.15, and 0.05 mm·yr^–1^ in the Huaibei region, the Sunan region, and the entire province, respectively (Table 2). On an annual scale during 1990–2019, a significant increasing trend (*p*< 0.05) of the annual ET_o_ with a rate of 3.02 mm·yr^–1^ was seen in the Sunan region, while the rate of increase in the annual ET_o_ in the Huaibei and Jianghuai regions was only 0.66 and 0.59 mm·yr^–1^, which caused the annual ET_o_ of the entire province to increase insignificantly at a rate of only 0.98 mm·yr^–1^ during 1990–2019.

Different from the ET_o_, which generally displayed a negative trend during 1960–1989 and a positive trend during 1990–2019, the seasonal and annual ET_o_ showed various changing trends in each subregion and throughout the entire province during 1960–2019 (Table 2). Specifically, the spring ET_o_ increased in all the subregions at a rate ranging from 0.04 mm·yr^–1^ in the Huaibei region to 1.04 mm·yr^–1^ in the Sunan region (*p*< 0.001) during 1960–2019, which consequently caused the spring ET_o_ of the entire province to increase significantly (*p*< 0.01) at a rate of 0.53 mm·yr^–1^. Quite the contrary, the summer ET_o_ showed a decreasing trend in all the subregions at a rate ranging from –0.82 mm·yr^–1^ in the Huaibei region (*p*< 0.001) to –0.015 mm·yr^–1^ in the Sunan region, which therefore caused the summer ET_o_ of the entire province to decrease significantly (*p*< 0.05) at a rate of –0.53 mm·yr^–1^ during 1960–2019. Moreover, Table 2 demonstrates that, during 1960–2019, the ET_o_ decreased at a rate of –0.28 and –0.16 mm·yr^–1^ (*p*< 0.05) in the Huaibei region and at a rate of –0.05 and –0.01 mm·yr^–1^ in the entire province in autumn and in winter, respectively; nonetheless, it rose in autumn and in winter respectively at a rate of 0.01 and 0.09 mm·yr^–1^ in the Jianghuai region and at a rate of 0.36 (*p*< 0.01) and 0.09 mm·yr^–1^ in the Sunan region. On an annual scale, the ET_o_ enlarged at a rate of 0.08 and 1.32 mm·yr^–1^ (*p*< 0.05) in both the Jianghuai and Sunan regions respectively during 1960–2019; however, the annual ET_o_ in the Huaibei region decreased significantly at a rate of –1.37 mm·yr^–1^ (*p*< 0.001), which led to the dominant negative trend of the annual ET_o_ with a rate of –0.15 mm·yr^–1^ throughout the entire province during 1960–2019.

In general, except for the spring ET_o_ showing a positive trend in the Jianghuai region, in the Sunan region, and throughout the entire province during 1960–1989, the ET_o_ of the province of Jiangsu declined in the first 30-year period in the other seasons and throughout the year; the highest rate of reduction in ET_o_ for each subregion and in the entire province was all seen in summer; the lowest rate of reduction in ET_o_ for Jianghuai region was observed in winter, while the Huaibei region, the Sunan region, and the entire province had the lowest rate of reduction in the autumn ET_o_ (Table 2). Additionally, during 1990–2019, the seasonal and annual ET_o_ generally followed a positive trend in the province of Jiangsu with the highest increasing trend in spring and the lowest increasing trend in winter. During the whole study period from 1960 to 2019, the ET_o_ in the Huaibei region and in the entire province decreased in all the seasons and the whole year except for spring in which the ET_o_ showed a positive trend (Table 2). During 1960–2019, in the two other subregions, we observed an increasing trend in all the seasons and the whole year, except for summer with a negative trend. Moreover, the various changing trends of the ET_o_ on seasonal and annual scales throughout this province can impact on the spatiotemporal characteristics of the “evaporation paradox” in the corresponding seasons and subregions.

### Spatiotemporal characteristics of “evaporation paradox” in province of Jiangsu

**Fig 2:**
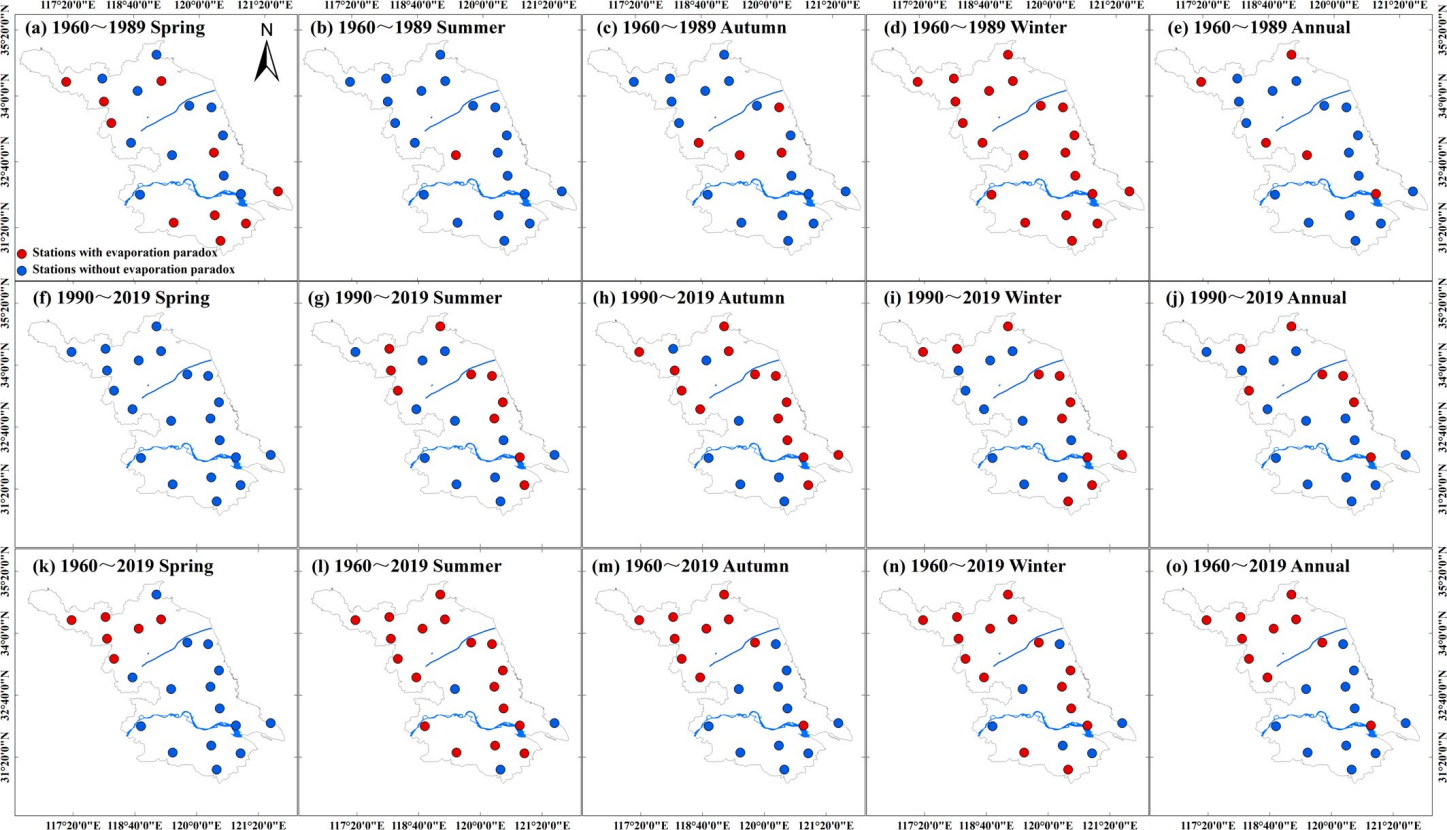
Spatial distributions of the sites with the “evaporation paradox” on seasonal and annual scales during 1960–1989, 1990–2019, and 1960–2019 throughout the province of Jiangsu; the solid red and blue circles indicate the presence and absence of the “evaporation paradox”, respectively.

During 1960–1989, the “evaporation paradox” was observed in 57.1% and 100% of the sites of the Huaibei region in spring and in winter respectively (Fig 2A and 2D), which consequently led to the “evaporation paradox” in these two seasons in the entire Huaibei region (Table 3). Furthermore, the “evaporation paradox” was found in all the sites of the Jianghuai region in winter during 1960–1989 (Fig 2D), while we observed the “evaporation paradox” in only 22.2%, 11.1%, 44.4%, and 33.3% of the sites of this subregion in spring, in summer, in autumn, and throughout the year respectively (Fig 2A–2C and 2E). Correspondingly, according to Table 3, the “evaporation paradox” existed only in winter in the entire Jianghuai region during 1960–1989. Consistent with the Huaibei region, the “evaporation paradox” also happened in spring and in winter in the whole Sunan region (Table 3); in fact, 80% and 100% of the sites of the “evaporation paradox” were in these two seasons during 1960–1989 (Fig 2A and 2D). In the first 30-year period, 100% of the sites had the “evaporation paradox” (Fig 2D) in the entire province in winter (Table 3), while less than 50% of the sites experienced the “evaporation paradox” in the entire province in the other seasons in the whole year (Fig 2A–2C and 2E).

**Table 3:** The existence of the “evaporation paradox” in the Huaibei (HB), Jianghuai (JH), and Sunan (SN) regions and throughout the entire province of Jiangsu on seasonal and annual scales in the three study periods of 1960–1989, 1990–2019, and 1960–2019.

Period	Zone	Spring	Summer	Autumn	Winter	Annual
1960–1989	HB	○	×	×	○	×
	JH	×	×	×	○	×
	SN	○	×	×	○	×
	Whole	×	×	×	○	×
1990–2019	HB	×	×	○	×	×
	JH	×	○	○	○	×
	SN	×	×	×	×	×
	Whole	×	×	○	×	×
1960–2019	HB	×	○	○	○	○
	JH	×	○	×	×	×
	SN	×	○	×	×	×
	Whole	×	○	○	○	○

Note: ○ and × indicate the existence or the absence of the “evaporation paradox” respectively.

During 1990–2019, the “evaporation paradox” only existed in autumn for the entire Huaibei region (Table 3) with 71.4% of the sites of “evaporation paradox” (Fig 2H). However, 55.5%, 88.8%, and 66.6% of the sites of the “evaporation paradox” happened in summer, autumn, and winter respectively in the whole Jianghuai region (Table 3) during 1990–2019 (Fig 2G–2I). For the southern Sunan region, during 1990–2019, there were few sites with the “evaporation paradox” (Fig 2F–2J), which thus caused the “evaporation paradox” not to exist in the whole Sunan region on both seasonal and annual scales (Table 3). As Fig 2H shows, it was consistent with the Huaibei region where only 66.6% of the sites had the “evaporation paradox” in autumn in the entire province (Table 3).

According to Fig 2K–2O and Table 3, except for spring, 100% of the sites of the whole Huaibei region experienced the “evaporation paradox” in the other seasons in the whole year during 1960–2019. However, the “evaporation paradox” was found only in summer for the entire Jianghuai and Sunan regions in 1960–2019 with 77.7% and 80% of the sites of “evaporation paradox”, respectively (Fig 2L). For the entire province during 1960–2019, the “evaporation paradox” was found in 85.7%, 61.2%, and 66.6% of the sites respectively in summer, autumn, and winter (Fig 2L–2N), which therefore led to the “evaporation paradox” in the entire province in these three seasons (Table 3). On an annual scale during 1960–2019, although the “evaporation paradox” existed in only 47.5% of the sites (mostly located in the northern Huaibei region) of the entire province (Fig 2O), the significantly decreased annual ET_o_ in the Huaibei region dominated the negative trend of the annual ET_o_ (Table 2) and resulted in the “evaporation paradox” in the entire province of Jiangsu annually (Table 3).

According to Table 3, during 1960–1989, the “evaporation paradox” was observed in both spring and winter in the whole Huaibei and Sunan regions but only in winter both in the whole Jianghuai region and in the entire province of Jiangsu. In the second 30-year period, the “evaporation paradox” was only observed in autumn both in the whole Huaibei region and in the entire province of Jiangsu. However, we detected the “evaporation paradox” in the whole Jianghuai region in more seasons, namely summer, autumn, and winter, during 1990–2019. In the whole Huaibei region and the entire province of Jiangsu, the “evaporation paradox” was found in all the seasons and in the whole year except for spring during 1960–2019. Moreover, the “evaporation paradox” was only found in summer in the whole Huaibei and Sunan regions during 1960–2019.

### Causes analysis of “evaporation paradox” in province of Jiangsu

#### Annual original and detrended meteorological variables in the entire province

**Fig 3:**
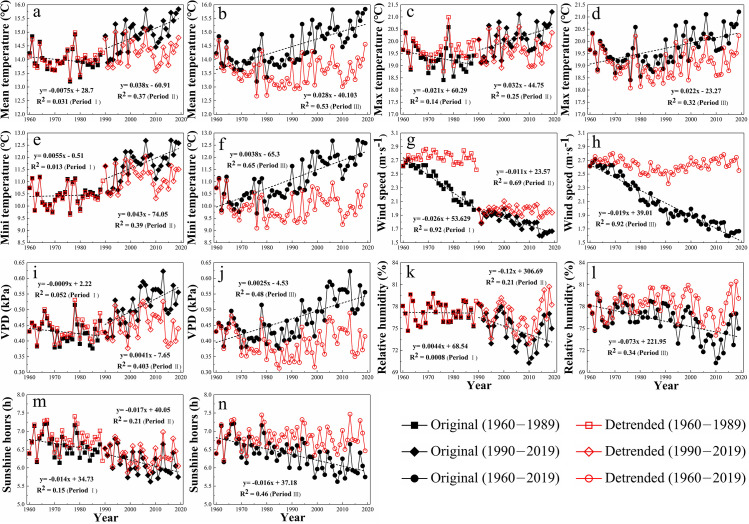
The annual original and detrended meteorological variables, including (a,b) *T*_mean_, (c,d) *T*_max_, (e,f) *T*_min_, (g,h) WS, (i,j) VPD, (k,l) RH, and (m,n) SH throughout the entire province of Jiangsu by using the detrending method; Periods I, II, and III represent 1960–1989, 1990–2019, and 1960–2019 respectively.

The annual original and detrended meteorological variables, including *T*_mean_, *T*_max_, *T*_min_, WS, VPD, and RH of the entire province were compared during 1960–1989, 1990–2019, and 1960–2019 (Fig 3). During 1960–1989, only the original annual *T*_min_ and RH showed a slight increasing trend, which led to the lower detrended annual *T*_min_ and RH in the entire province (Fig 3E and 3K). The five other meteorological variables all decreased during 1960–1989 and had a higher detrended value on an annual scale (Fig 3A, 3C, 3G, 3I and 3M). It was obvious that the largest difference was seen between the annual original data and the annual detrended data on the wind speed in the entire province during 1960–1989 (Fig 3G). In the second 30-year period, the annual original VPD and the annual original three air temperature variables all showed a positive trend, which led to the lower annual detrended values of these variables (Fig 3A, 3C, 3E and 3I). However, the annual detrended WS, RH, and SH of the entire province were higher than the annual original ones which exhibited decreasing trends during 1990–2019 (Fig 3G, 3K and 3M). It was noticed that the difference between the annual original VPD and the annual detrended VPD in the entire province was larger compared to all the other variables during 1990–2019 (Fig 3I).

Similar to the situation in the period from1990 to 2019, we observed an increasing trend of the annual VPD and the three air temperature variables in the entire province during 1960–2019, which consequently resulted in the lower annual detrended VPD and the lower three air temperature variables (Fig 3B, 3D, 3F and 3J). The three other variables also showed a decreasing trend and induced a higher annual detrended WS, RH, and SH in the province of Jiangsu during 1960–2019 (Fig 3H, 3L and 3N). Furthermore, we found that the difference in the annual original *T*_mean_, *T*_min_, WS, and VPD and the annual detrended ones was more significant compared to the other meteorological variables (Fig 3B, 3D, 3F, 3H, 3J, 3L and 3N), which indicated that the variation in these variables could impact on the trend of the ET_o_ in the entire province to a greater extent during 1960–2019. According to the example of the annual data in the entire province (Fig 3) and the changing trend of each meteorological variable on both seasonal and annual scales, we could distinguish the difference between each original meteorological variable and the detrended one on various spatiotemporal scales.

#### Contributions of meteorological variables to trends of ETo in entire province on annual scale

**Fig 4:**
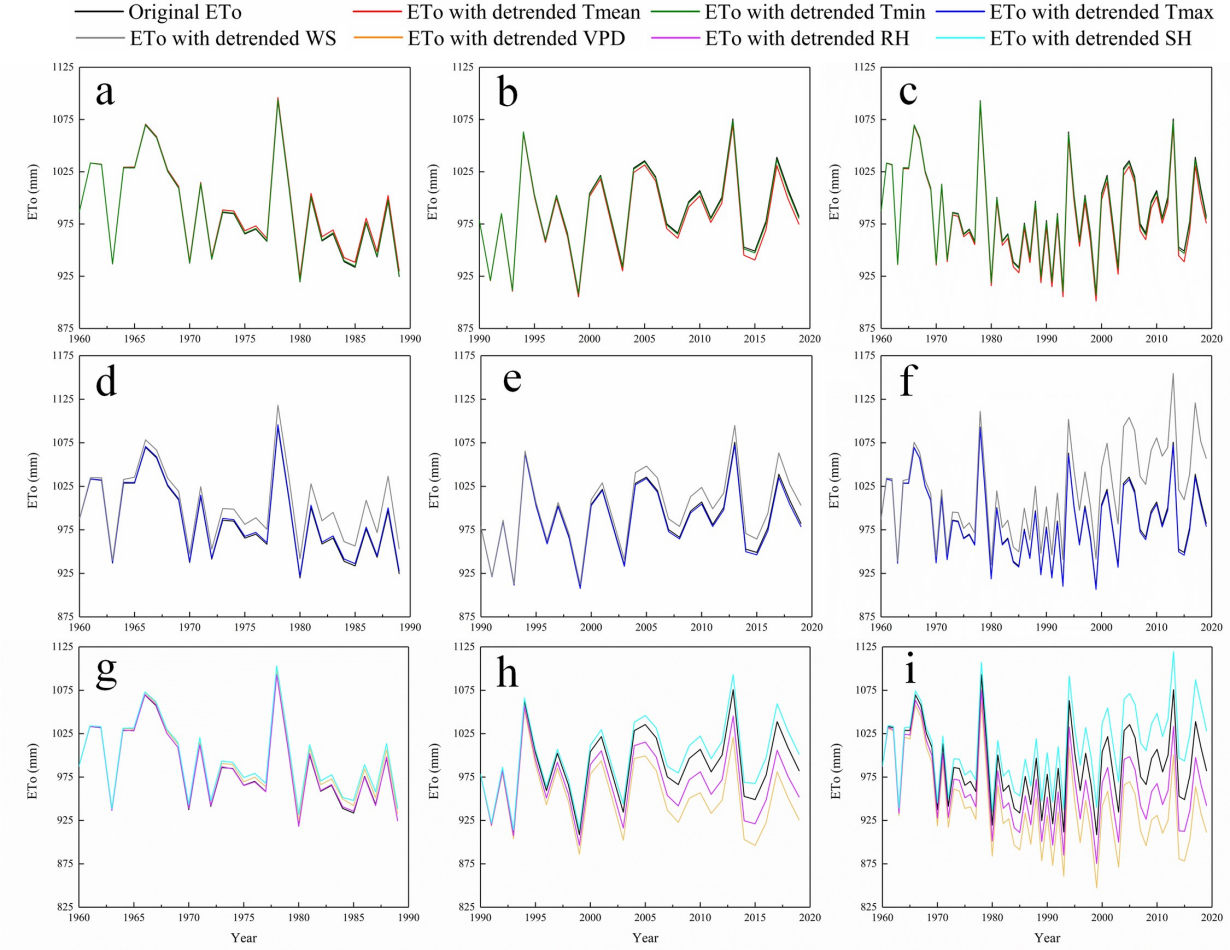
The original annual ET_o_ and the annual one recalculated based on the detrended (a,b,c) *T*_mean_ and *T*_min_; (d,e,f) *T*_max_ and WS; and (g,h,i) VPD, RH, and SH during 1960–1989, 1990–2019, and 1960–2019 throughout the entire province of Jiangsu.

The annual original ET_o_ and the recalculated one with each detrended variable in the entire province during 1960–1989, 1990–2019 and 1960–2019 are delineated in Fig 4 as an example so as to differentiate the original ET_o_ from the recalculated one. In the first 30-year period, the annual ET_o_ recalculated using the detrended RH and three air temperature variables was very close to the annual original ET_o_ (Fig 4A, 4D and 4G), which indicated that the variation in the annual RH and three air temperature variables had little impact on the changed annual ET_o_ in the entire province during 1960–1989. However, the variation of the other variables had a more significant impact on the trend of the annual ET_o_; in fact, the largest negative difference was seen between the annual original ET_o_ and the one recalculated based on the detrended WS, followed by the one recalculated using the SH and the VPD (Fig 4D and 4G). In the entire province during 1990–2019, the annual ET_o_ values recalculated based on the detrended VPD, RH, and three air temperature variables were all lower than the annual original ET_o_ (Fig 4B, 4E and 4H), while the annual ET_o_ values recalculated using the detrended WS and SH were found to be larger than the annual original ET_o_ (Fig 4E and 4H). Obviously, the changes in the annual air humidity variables (VPD and RH) dominated the positive trend of the annual ET_o_ in the entire province during 1990–2019; indeed, there was a larger difference between the annual original ET_o_ and the annual ET_o_ recalculated based on the detrended VPD and RH (Fig 4E and 4H).

Similar to the second 30-year period, in the entire province of Jiangsu during 1960–2019, only the annual ET_o_ values recalculated using the detrended WS and SH were found to be larger than the annual original one (Fig 4F and 4I), and the annual ET_o_ values recalculated based on the other detrended variables were all lower than the annual original one (Fig 4C and 4F). Furthermore, the largest difference was observed between the annual original ET_o_ and the one recalculated using the detrended VPD, followed by the one recalculated based on the WS, the RH, and the SH, while the annual ET_o_ values recalculated using the detrended air temperature variables were very close to the annual original one (Fig 4C, 4F and 4I). This indicated that the variations in the annual VPD, WS, RH, and SH, rather than the air temperature variables, dominated the trend of the annual ET_o_ in the entire province during 1960–2019.

#### Quantitative analysis of causes of “evaporation paradox” during 1960–1989

**Fig 5:**
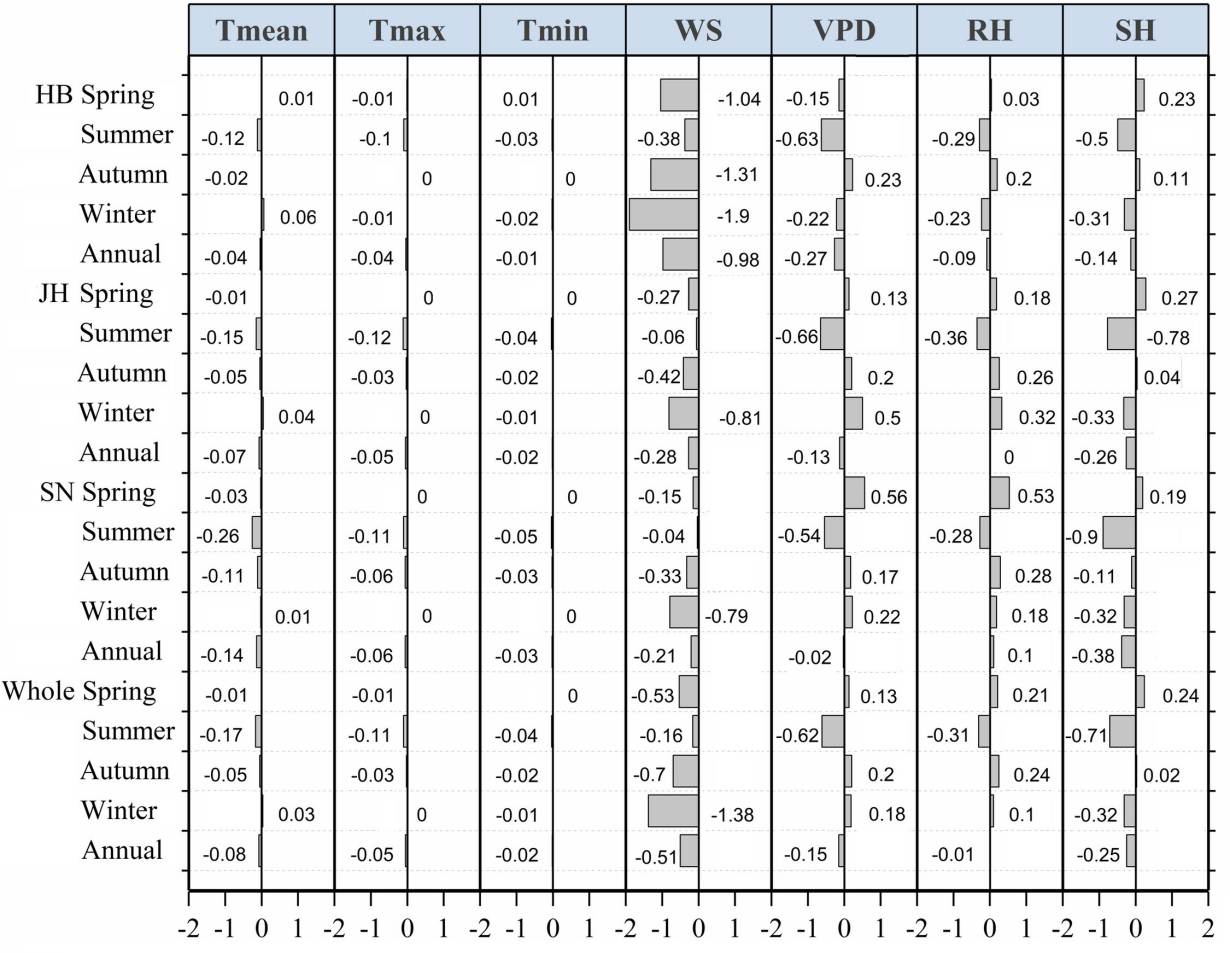
Comparison of indicator *R* for identifying the contribution of each meteorological variable to the trend of the ET_o_ on seasonal and annual scales in the Huaibei (HB), Jianghuai (JH), and Sunan (SN) regions and throughout the entire province of Jiangsu during 1961–1989. The indicator *R*, computed by the original ET_o_ and recalculated ET_o_ with each detrended meteorological variable, is for quantifying the contribution of a change in the certain variable to ET_o_ trend. *R* >0, *R* <0, and *R* = 0 separately denote positive, negative, and no contribution of this variable to the change of ET_o_. A larger value of *|R|* denotes that the variation in this variable affects the ET_o_ to a greater extent.

In the Huaibei region during 1960–1989, contrary to the increased *T*_mean_, the decreased wind speed was the main reason for the reduced ET_o_, which induced the “evaporation paradox” in spring and in winter (Fig 5 and Table 3). Moreover, the decreased WS and SH overwhelmed the positive impacts of the increased VPD and the decreased RH, leading to the reduced ET_o_ (Fig 5) and the “evaporation paradox” in winter in the Jianghuai region, in the Sunan region, and throughout the entire province during 1960–1989 (Table 3). It was noticed that the reduced *T*_mean_ and the increased ET_o_—mainly caused by the increased VPD and the reduced RH—led to the “evaporation paradox” in the Sunan region in spring during 1960–1989 (Fig 5 and Table 3). In summer, in autumn, and throughout the whole year, the “evaporation paradox” was not observed in any subregions and in the entire province during 1960–1989 (Table 3). Specifically, the observed decreasing trend of *T*_mean_ was accompanied by the reduced ET_o_ that was dominated by the decreased WS in autumn during 1960–1989 (Fig 5). In summer and throughout the whole year in the first 30-year period, both the *T*_mean_ and the ET_o_ followed a decreasing trend, but the latter was dominated by the negative impacts of the changes in the other variables, including the WS, VPD, RH, and SH (Fig 5). Generally, the sharply reduced WS dominated the negative trend of the ET_o_ and thus gave rise to the “evaporation paradox” in spring and in winter in the Huaibei region and only in winter both in the two other subregions and in the entire province during 1960–1989 (Fig 5 and Table 3). In the spring of the Sunan region during 1960–1989, the “evaporation paradox” was expressed by the decreased *T*_mean_ and the increased ET_o_ dominated by the positive effects of the variations in the VPD and the RH.

#### Quantitative analysis of causes of “evaporation paradox” during 1990–2019

**Fig 6:**
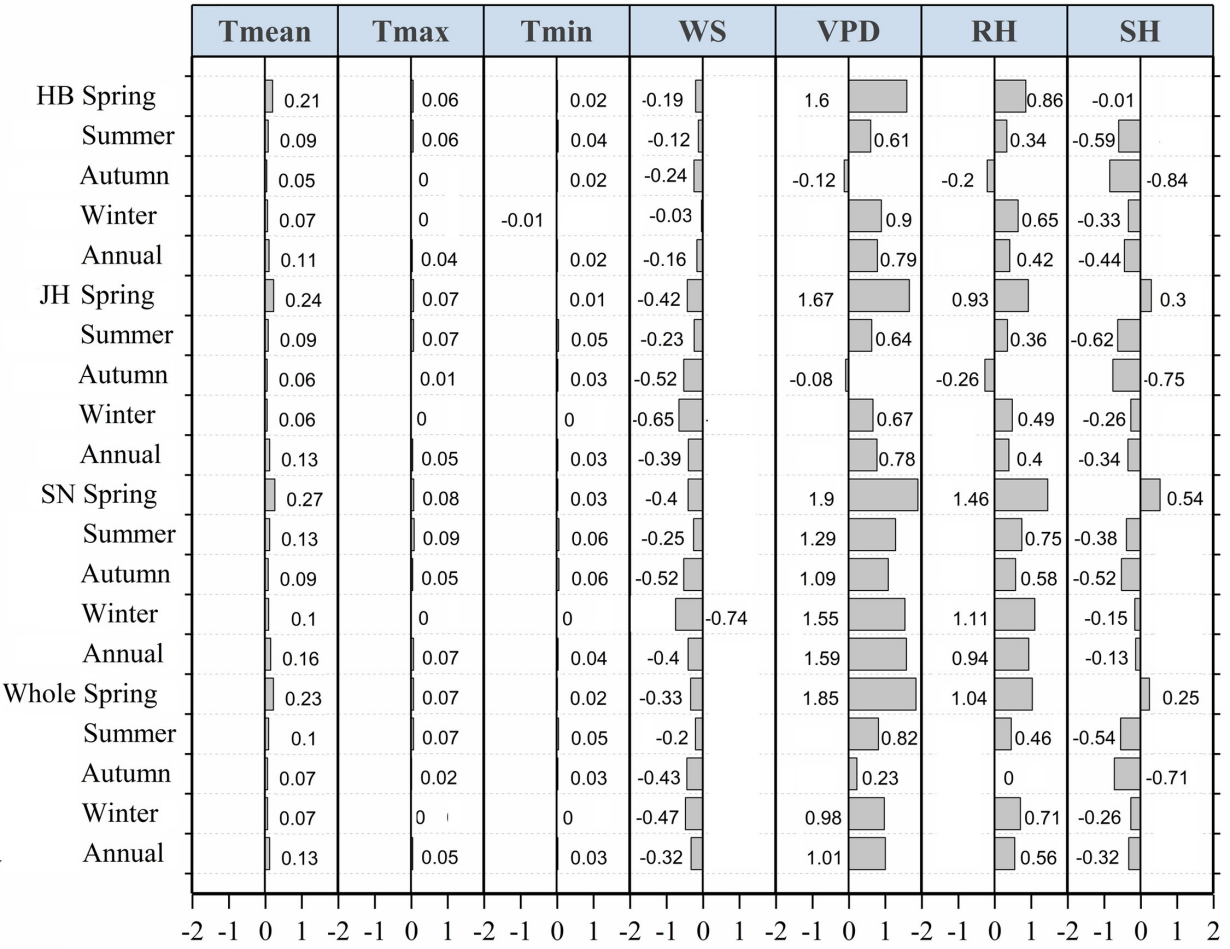
Comparison of indicator *R* for identifying the contribution of each meteorological variable to the trend of the ET_o_ on seasonal and annual scales in the Huaibei (HB), Jianghuai (JH), and Sunan (SN) regions and throughout the entire province of Jiangsu during 1990–2019. The indicator *R*, computed by the original ET_o_ and recalculated ET_o_ with each detrended meteorological variable, is for quantifying the contribution of a change in the certain variable to the ET_o_ trend. *R* >0, *R* <0, and *R* = 0 separately denote positive, negative, and no contribution of this variable to the change of ET_o_. A larger value of *|R|* denotes that the variation in this variable affects the ET_o_ to a greater extent.

During 1990–2019, the air temperature variables showed an increasing trend and resulted in a positive impact on the trend of the ET_o_ in the province of Jiangsu (Fig 6). However, similar to 1960–1989, the impact of the changes in the air temperature variables was much smaller than that of the other variables during 1990–2019 (Fig 6). In spring, the significant positive impact of the changes in the VPD and the RH gave rise to the increasing trend of the ET_o_, which was consistent with the increased *T*_mean_ (“no evaporation paradox”) in all the subregions and in the entire province during 1990–2019 (Fig 6). We observed that the situations in summer and winter were similar during 1990–2019; in fact, the variations in the VPD and the RH dominated the increased ET_o_ in all the subregions and throughout the entire province except for the Jianghuai region in which the decreased SH and WS dominated the reduced ET_o_ (Fig 6) and caused the “evaporation paradox” (Table 3). However, the reduced SH contributed more in summer, and the decreased WS contributed more in winter in the Jianghuai region during 1990–2019 (Fig 6). For the reduced ET_o_ giving rise to the “evaporation paradox” in autumn in the Huaibei region, in the Jianghuai region, and in the entire province during 1990–2019 (Tables 2 and 3), the most likely dominating factor was considered to be the decreased WS and SH, with a greater negative contribution of the decreased SH (Fig 6). On an annual scale, the impacts of the decreased WS and SH were overwhelmed by the positive impact of the changes in the VPD, RH, and *T*_mean_, leading to the increased ET_o_ which matched the warming trend in all the subregions and throughout the entire province during 1990–2019 (Fig 6). In general, the decreased SH was the main reason for the reduced ET_o_ and thus led to the “evaporation paradox” in both summer and autumn in the Jianghuai region and only in autumn both in the Huaibei region and in the entire province during 1990–2019 (Fig 6 and Table 3). However, the decreased WS dominated the reduced ET_o_ and the “evaporation paradox” in winter in the Jianghuai region during 1990–2019 (Fig 6 and Table 3).

#### Quantitative analysis of causes of “evaporation paradox” during 1960–2019

**Fig 7:**
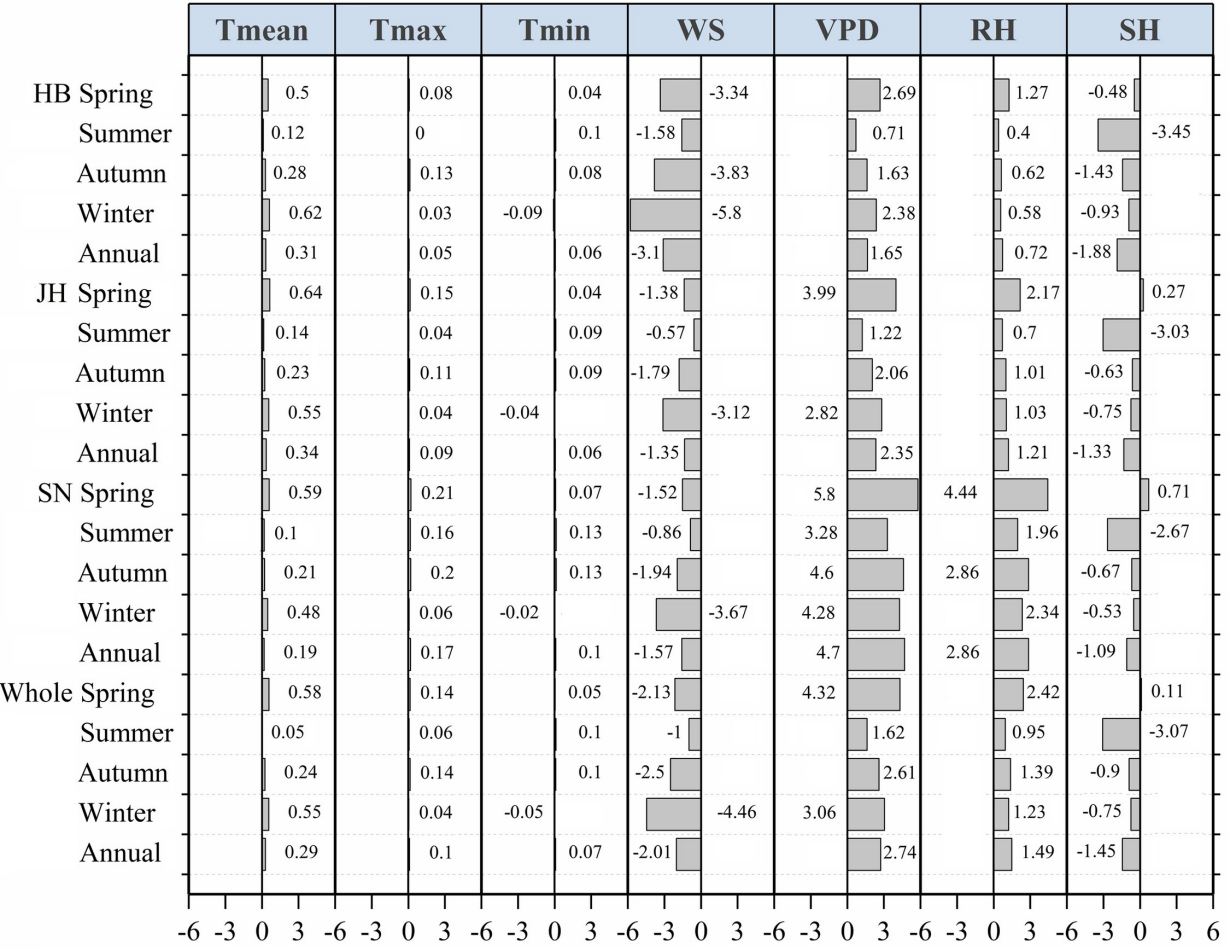
Comparison of indicator *R* for identifying the contribution of each meteorological variable to the trend of the ET_o_ on seasonal and annual scales in the Huaibei (HB), Jianghuai (JH), and Sunan (SN) regions and throughout the entire province of Jiangsu during 1960–2019. The indicator *R*, computed by the original ET_o_ and recalculated ET_o_ with each detrended meteorological variable, is for quantifying the contribution of a change in the certain variable to the ET_o_ trend. *R* >0, *R* <0, and *R* = 0 separately denote positive, negative, and no contribution of this variable to the change of ET_o_. A larger value of |*R*| denotes that the variation in this variable affects the ET_o_ to a greater extent.

Similar to the second 30-year period, the variation of the air temperature variables caused a positive, but negligible, influence on the trend of the ET_o_ throughout the province of Jiangsu during 1960–2019 (Fig 7). Consistent with the increased *T*_mean_, the changes in the VPD and the RH overwhelmed the negative impacts of the reduced WS and SH, leading to the increased ET_o_ in all the subregions and throughout the entire province in spring during 1960–2019 (Fig 7). Further, the decreased WS and SH (especially the latter) were considered to be the main reason for the reduced ET_o_ giving rise to the “evaporation paradox” in all the subregions and throughout the entire province in summer during 1960–2019(Fig 7). In autumn, in winter, and throughout the whole year during 1960–2019, the “evaporation paradox” was found both in the Huaibei region and in the entire province, while the reduced ET_o_ was mainly caused by the decreased WS and SH, with a greater impact of the former (Fig 7 and Table 3). Generally, the decreased WS and SH contributed to the reduced ET_o_ and induced the “evaporation paradox” in summer in all the subregions and throughout the entire province with a more remarkable negative impact of the decreased SH during 1960–2019 (Table 3 and Fig 7). Regarding the “evaporation paradox” in autumn, in winter, and throughout the whole year in the Huaibei region and throughout the entire province during 1960–2019, the decreased WS and SH dominated the reduced ET_o_, but the former had a more significant effect(Table 3 and Fig 7).

## Discussion

### Spatiotemporal characteristics of “evaporation paradox” in province of Jiangsu

This work examined and found the staged variations of the *T*_mean_ and the ET_o_ on seasonal and annual scales throughout the province of Jiangsu during the past 60 years and (Tables 1 and 2). In general, the *T*_mean_ showed an insignificant decreasing trend in this province during 1960–1989 (Table 1), which was similar to some previous studies with a study period prior to 1990 [15, 20, 24, 26, 51]. However, the *T*_mean_ of the province of Jiangsu during 1990–2019 followed a significant increasing trend (*p*< 0.001) and thus dominated the significantly increased *T*_mean_ (*p*< 0.001) during the whole study period from 1960 to 2019 (Table 1). Similarly, a warming trend has also been reported in many regions worldwide [5, 6, 16, 20, 49, 52, 53] and globally [1] with a longer study period including the last three decades. Furthermore, in addition to the higher greenhouse gas emissions and the expanded cloudiness worldwide [6, 54], the regionally rapid urbanization and industrialization of the province of Jiangsu have also contributed to the positive trend of the air temperature in the last three decades [26, 55–57].

Regarding the view proposed by Xing et al. [8] that most previous studies have chiefly focused on the general trends of the ET_o_ without taking account of the abrupt changes [6, 54], we examined the changing trends of the ET_o_ in the province of Jiangsu during 1960–2019 and found that the change points of both the seasonal ET_o_ and the annual one happened almost in 1990 (Table 2). Similar to our results, some previous works have also reported that the ET_o_ first decreased but then increased with a change point almost in 1990 in the Hai River basin [30], in the arid region of northern China [24], and in China as a whole [6, 24]; the study period of all the works were longer than 50 years. However, Cong et al. [23] and Ma et al. [15] investigated the changing trends of the ET_o_ in China and in the Yellow River basin respectively, and both reported the change points of the ET_o_ in the first half of 1980s. The earlier change point proposed by Cong et al. [23] was chiefly due to the study period from 1956 to 2005, which is 15 years ahead of this work. Furthermore, the change point in 1980 reported by Ma et al. [15] was artificially selected based on the first comprehensive evaluation period of water resources, organized by Chinese government and ended in 1980.

Different from some previous works reporting on the “evaporation paradox” with a study period of longer than 40 years [15–17, 27], the staged variations of the *T*_mean_ and the ET_o_ during 1960–2019 have resulted in the unique characteristics of the “evaporation paradox” in the province of Jiangsu (Table 3). On a seasonal scale, the “evaporation paradox” generally appeared in winter during 1960–1989, in autumn during 1990–2019, and in summer during 1960–2019 in this province (Table 3). On a regional scale, the “evaporation paradox” occurred more frequently in the northern Huaibei region and regulated the spatiotemporal characteristics of the “evaporation paradox” in the entire province of Jiangsu during 1960–2019 (Fig 2 and Table 3). Similarly, some previous studies reported an “evaporation paradox” accompanied by a decreased ET_o_ and an increased *T*_mean_ within China [15, 17, 24, 29, 30] and in other countries or regions [5, 6, 27, 58–60]. However, we found another form of the “evaporation paradox” expressed by a decreased *T*_mean_ and an increased ET_o_ in spring in the Sunan region during 1960–1989 (Table 3). This form of the “evaporation paradox” was also reported in the Yellow River basin of northern China during 1960–1979 [15] and in the province of Yunnan in southwestern China during 1981–1990 [20]. In general, these two forms of the “evaporation paradox” were both caused by the negligible impact of the changed *T*_mean_ and the more significant impacts of the decreased SH or WS on the reduced ET_o_ and the increased atmospheric demand for the increased ET_o_ [15, 17, 24, 26, 27, 29].

### Impacts of meteorological variables on trends of ET_o_

In warming climate, there has been a long-term expectation that the ET_o_ is largely affected by the increased air temperature and thus has shown an increasing trend in the past decades [19]. However, for the three periods studied herein, the impact of all the changed air temperature variables was found to be much smaller than that of the other variables in the province of Jiangsu (Figs 5–7), which was consistent with the studies on many regions within China [16–18, 26, 29, 32, 48] and even with those on the entire country [19, 23]. However, Zhang et al. [24] and Fan et al. [25] found an increased ET_o_ in the mountain plateau zone of western China during 1956–2015 and in China as a whole during 1993–2011. They also proposed that the increased ET_o_ was caused by the increased *T*_min_ and *T*_mean_ in the mountain plateau zone of western China and in China as a whole, respectively. The contradiction between the conclusions of these two works and ours likely arises from the different underlying surfaces, the study durations, and the evaluation methods [24, 25].

The wind speed, as an important aerodynamic term, was the only variable making negative impacts on both seasonal and annual trends of the ET_o_ during all the three study periods in the province of Jiangsu (Figs 5–7). In general, the reduced wind speed dominated the decreasing trend of the ET_o_ in autumn, in winter, and throughout the whole year in all the subregions and throughout the entire province during 1960–1989 (Table 2 and Fig 5) and in the Huaibei region and throughout the entire province during 1960–2019 (Table 2 and Fig 7). Similarly, a global decline in the wind speed was also reported by McVicar et al. (2012) [35], and some previous studies attributed the reduced ET_o_ to the decreased wind speed on a regional scale as well [15, 16, 23, 26, 27]. However, in Bei Dagan in the Central District of Israel with enhanced air pollution and urbanization, Cohen et al. [61] found an increasing trend of the wind speed on a site scale, which contributed to the increased ET_o_ during 1964–1998. In our work, the sunshine hour, which limits the amount of energy available to vaporize water [39], was a variable dominating the decreased ET_o_ in all the subregions and throughout the entire province in autumn during 1990–2019 and in summer during 1960–1989 and 1960–2019 (Figs 5 and 6). Similarly, the sunshine hour and its dominant solar radiation were found to largely impact the trends of the ET_o_ in the Hai River basin during 1960–1989 [30], in the northern and southern regions of Qinling mountains during 1960–2011 [62], in the subtropical monsoon zone during 1956–2015 [25], and in the humid region of China during 1960–1993 [24].

Regarding the increased ET_o_ observed in this study, the increased VPD and the decreased RH were considered to be the main factors which overwhelmed the negative impacts of the decreased WS and SH (Figs 5–7). Moreover, relative humidity, which only indicates the degree of the moisture content of the atmosphere, was found to follow a decreasing trend and dominated the increased ET_o_ in studies prior to 2015 [32–34]. However, Novick et al. [63] proposed a view that the atmospheric demand for water was directly related to the VPD, which was found as the key factor in the increased ET_o_ in this study (Figs 6 and 7). Similarly, the increased VPD was found to be an important factor affecting the increased ET_o_ in the arid region of northern China [24], in the province of Jilin in northeastern China [17], in the Qinhuai River basin [26], and in the Huai River basin in eastern China [64]. The sharply strengthening atmospheric demand and the inhibited evaporation related to rapid urbanization were the important factors influencing the trends of the ET_o_ [65] and should be investigated further in the future.

### Deficiencies of this study

This work analyzed the spatiotemporal characteristics of the “evaporation paradox” and identified the dominating meteorological variables controlling it in the three subregions and throughoutthe entire province of Jiangsu during 1960–2019. However, its main drawbacks include:

- Although the ET_o_ is a term that closely resembles the maximum possible evapotranspiration [39] and has been widely used in similar studies [6, 8, 15, 16, 23, 26, 27], it is still necessary to combine the observed ET_pan_ and the simulated potential evapotranspiration as the two other evaluation variables of the evaporation capacity so as to discuss the “evaporation paradox” in future works.
- In addition to the seven conventionally observed meteorological variables that directly impact on the trend of the ET_o_, some other parameters such as cloud cover and aerosol optical depth can control the variations in the ET_o_ [17] and thus should be further investigated.

## Conclusions

This study separately quantified the individual impact of seven meteorological variables on the trends of the ET_o_ and identified the dominant meteorological factors affecting the “evaporation paradox” on both seasonal and annual scales in the three subregions and throughout the entire province of Jiangsu during the past 60 years. The research on the period from 1960 to 2019 demonstrated that the change point of the different meteorological variables and that of the ET_o_ were chiefly found almost in 1990, so this study was conducted in the three periods: from 1960 to 1989, from 1990 to 2019, and from 1960 to 2019. Prior to 1990, the significantly decreased wind speed was responsible for the reduced ET_o_ and the “evaporation paradox” in spring and winter in the Huaibei region and only in winter in the two other subregions and throughout the entire province. Furthermore, the “evaporation paradox” in spring in the Sunan region was expressed by the decreased daily mean temperature and the increased ET_o_ dominated by the increased vapor pressure deficit and the reduced relative humidity during 1960–1989. During the period from 1990 to 2019, the decreased wind speed was also the main cause of the reduced ET_o_ and the “evaporation paradox” in winter in the Jianghuai region. However, in the second 30-year period, the decline in the sunshine hour was found to be the dominating factor controlling the decreased ET_o_ which gave rise to the “evaporation paradox” in summer in the Jianghuai region and in autumn in the Huaibei region, in the Jianghuai region, and throughout the whole province. During the entire study period from 1960 to 2019, the decreased sunshine hour dominated the reduced ET_o_ and thus induced the “evaporation paradox” in summer in all the subregions and throughout the entire province. However, the decrease in the wind speed controlled the reduced ET_o_ and the “evaporation paradox” in autumn, in winter, and throughout the whole year both in the Huaibei region and in the entire province during 1960–2019.

Generally, the variations in the wind speed and the sunshine hour, rather than the air temperature, largely impacted on the trends of the ET_o_ and induced the “evaporation paradox” observed in some specific seasons and regions mentioned above. Further, we also found that the sharply increased vapor pressure deficit contributed to the increased ET_o_ in the province of Jiangsu, especially in the Sunan region during 1990–2019. The significantly increased atmosphere demand, that is, the increased vapor pressure deficit, also accelerated the hydrological cycle [8] and raised the pressure of water resource management in this province. In addition to the climate change, the “evaporation paradox” is a reaction to the active human activities after 1990 such as increased air pollution [66], rapid industrialization, and urbanization [56, 65]. Therefore, the future researches addressing hydrological cycle processes and the environment should take the effects of both climate change and human activities into account.

## Supporting information

S1 Data (XLSX)
